# Perinatally diagnosed congenital craniopharyngiomas in the KRANIOPHARYNGEOM trials

**DOI:** 10.1530/EC-23-0294

**Published:** 2023-11-15

**Authors:** Julia Beckhaus, Svenja Boekhoff, Katrin Scheinemann, Freimut H Schilling, Gudrun Fleischhack, Gerhard Binder, Brigitte Bison, Torsten Pietsch, Carsten Friedrich, Hermann L Müller

**Affiliations:** 1 Department of Pediatrics and Pediatric Hematology/Oncology, University Children’s Hospital, Carl von Ossietzky University, Klinikum Oldenburg AöR, Oldenburg, Germany; 2 Division of Epidemiology and Biometry, Carl von Ossietzky University, Oldenburg, Germany; 3 Pediatric Hematology-Oncology Center, Children’s Hospital of Eastern Switzerland, St. Gallen, Switzerland; 4 Faculty of Health Sciences and Medicine, University of Lucerne, Lucerne, Switzerland; 5 Department of Pediatrics, McMaster Children’s Hospital and McMaster University, Hamilton, Ontario, Canada; 6 Department of Pediatrics, Division of Pediatric Hematology and Oncology, Children’s Hospital Lucerne, Lucerne, Switzerland; 7 Pediatrics III, University Hospital of Essen, Essen, Germany; 8 University Children’s Hospital, Pediatric Endocrinology, University Tübingen, Tübingen, Germany; 9 Diagnostic and Interventional Neuroradiology, Faculty of Medicine, University of Augsburg, Augsburg, Germany; 10 Institute of Neuropathology, DGNN Brain Tumor Reference Center, University of Bonn Medical Center, Bonn, Germany

**Keywords:** craniopharyngioma, congenital, neurosurgery, irradiation, quality of life

## Abstract

**Background:**

Craniopharyngiomas (CPs) are rare embryonic tumors. Clinical presentation and outcome of patients perinatally diagnosed with congenital CP (cCP) are not clear and refer mainly to a few case reports in the literature. The aim of this study was to analyze clinical presentation and outcome in patients with cCP.

**Study design:**

Three hundred and sixty-one patients diagnosed with adamantinomatous CP were recruited 2007–2022 in KRANIOPHARYNGEOM 2007/Registry 2019 and prospectively observed. In two cases, cCP was diagnosed prenatally and in one case on the second day of life. Pre- and perinatal diagnostic findings, postnatal evaluation, and therapeutic interventions and outcome in these three cases of cCP were analyzed.

**Results:**

All patients survived. One patient developed psychomotor retardation and a mild hemiparesis. Prenatal routine ultrasound examination led to the diagnosis of cCP. Tumor resection was performed during the early postnatal period (range: 11–51 days of age). Functional capacity, measured by Fertigkeitenskala-Münster-Heidelberg (FMH) was reduced in three and behavioral parameters, measured by the Strength and Difficulties Questionnaire (SDQ) were abnormal in two cases.

**Conclusion:**

cCP is a rare diagnosis with a prevalence of 0.83% in our study group. Compared to cases reported in the literature, the presented cases were treated immediately and had a better prognosis. Based on improvements of diagnostic and therapeutic techniques, prenatal diagnosis of cCP should lead to transfer prior to delivery of cCP patients to a specialized center for delivery and postnatal treatment of newborns with sellar masses by a multidisciplinary team to secure the improved prognosis of these patients.

**Significance statement:**

We previously reported that lower event-free survival rates after craniopharyngioma are associated with younger age at diagnosis. Perinatally diagnosed congenital craniopharyngiomas are very rare. This article presents three unique cases with congenital craniopharyngioma, comparing their diagnostics, therapy, and development. All three cases had surgery during the early postnatal period with sparing of the posterior hypothalamus. In each case, endocrinopathy was present at follow-up. Low functional capacity was reported in all cases and an abnormal total difficulties score in two cases. Compared to the literature, the presented cases had better prognosis in morbidity and mortality. This report and the review of the literature confirm the importance of a multidisciplinary approach in the diagnostic and treatment of the very rare condition of congenital craniopharyngioma.

## Introduction

Craniopharyngiomas (CPs) are rare, benign tumors (WHO grade I) (1) of the sellar region with an incidence of 0.5 to 2 new cases per million persons per year (2, 3, 4, 5). The age distribution at CP diagnosis has two peaks, one during childhood (5–9 years) and one in adults (55–69 years) (1, 6, 7). The adamantinomatous type is the most common nonneuroepithelial intracranial tumor in children (<18 years of age), accounting for around 5–11% of intracranial tumors in this age group, while the papillary type is almost only prevalent in adults (1). The diagnosis of CP in the fetal and neonatal periods is uncommon. Due to modern and noninvasive diagnostic techniques such as MRI and high-quality ultrasound screening during pregnancy, the incidence of tumors discovered during fetal life has increased (8).

CP is located in close proximity to the hypothalamus and the pituitary gland. Infiltration and displacement of both structures result in hormonal dysfunction (9). Depending on the size and location of the tumor, adverse side effects of CP can also include hydrocephalus and visual impairment (10). Not infrequently, children with CP show disturbances in their development and growth. Especially in CP patients with hypothalamic involvement (HI) and/or treatment-related hypothalamic lesion (HL), the body mass index (BMI) increases during the first year after surgery, frequently resulting in morbid obesity (11, 12, 13, 14, 15). Severe obesity increases the risk of cardiovascular complications and may result in a shorter life expectancy compared to a healthy population of the same age (16, 17, 18, 19). In contrast to other cancer survivors, children with childhood-onset CP suffer from severe long-term sequelae and need specialized care and a multidisciplinary approach to secure functional capacity in daily life and quality of life (13, 20).

We have previously reported on the negative effect of young age at diagnosis on prognoses and outcome after CP (21, 22). CPs are rarely diagnosed in the perinatal period. In our prospective observational studies KRANIOPHARYNGEOM 2007 and KRANIOPHARYNGEOM Registry 2019, we analyzed a cohort of 361 pediatric CP patients and detected three cases of congenital craniopharyngioma (cCP). We analyzed prenatal diagnostic findings, postnatal evaluation, and therapeutic interventions and outcome in these three cases of cCP.

## Patients and methods

### Patient cohorts and methods

Between 2007 and 2022, 361 (178 female/183 male) patients diagnosed with adamantinomatous CP at median age of 8.8 years (range: 0.01–18 years) were prospectively observed in the clinical trials/registries KRANIOPHARYNGEOM 2007 (Clinical Trial No. NCT01272622) (23) and in the KRANIOPHARYNGEOM Registry 2019 (Clinical Trial No. NCT04158284) and analyzed after a median follow-up interval of 4.2 years (range: 0.1–15.4 years). Eligibility criteria were: diagnosis of adamantinomatous CP confirmed by central pathological review at an age <18 years at CP diagnosis, and residing in Germany, Austria, Switzerland or Belgium.

Perinatally diagnosed CP was diagnosed in patients during the first 7 days of life. Prenatally diagnosed CP was present in fetus and diagnosed through routine ultrasonography.

### Neuroimaging

According to the KRANIOPHARYNGEOM 2007 and KRANIOPHARYNGEOM Registry 2019 protocols (24, 25, 26), cranial MRIs were performed at the time point of CP diagnosis and prospectively at 3-month intervals during the first year of follow-up after CP diagnosis. The neurological assessment (B.B.) regarding presurgical hypothalamic involvement (HI), tumor volume and location of CP, degree of surgical resection, and surgical hypothalamic lesions (HL) were published elsewhere (24, 25). The tumor volume of CP was calculated using the formula ½ (*A* × *B* × *C*) (aligned to the ellipsoid model: 4/3 π (*A*/2 × *B*/2 × *C*/2)), where *A*, *B*, and *C* are the maximum dimensions in the standard planes: axial (transverse, *A*), coronal (craniocaudal, *B*), and sagittal (anteroposterior, *C*) (26).

### Clinical parameters

In all CP patients, clinical and auxiological parameters were analyzed based on the protocols of the trials KRANIOPHARYNGEOM 2007 and the KRANIOPHARYNGEOM Registry 2019 (27). Body weight, body length, and head circumference were measured at birth. Percentiles were acquired from the age- and sex-related references of Voigt *et al.* (28) for each newborn patient with cCP. Furthermore, body weight, body height, and BMI were measured at last visit. BMI (w/h^2^; w = weight (kg), h = height (m)) was calculated as standard deviation score (s.d.s.) according to the age-related references of Rolland-Cachera *et al.* (29). For height and weight, *Z*-scores and respective percentiles were calculated using the age-related references (mean and standard deviation) of Prader *et al.* (30).

### Quality of life questionnaires

#### German daily life ability scale Fertigkeitenskala Münster-Heidelberg

To analyze functional capacity, we used the German daily life ability scale Fertigkeitenskala Münster-Heidelberg (FMH) at last visit (31). Based on 56 items, the FMH instrument measures the capacity for routine, daily life actions. FMH was normalized with 971 individuals (45% female), aged between 0 and 102 years. FMH scores have the format of age-dependent percentiles (32). The time for answering the FMH questionnaire is on average 4.5 min (31). In this study, the FMH was evaluated by parents.

#### The Strengths and Difficulties Questionnaire

The Strengths and Difficulties Questionnaire (SDQ) is a brief behavioral screening questionnaire for 2- to 17-year-old children (33). The SDQ comprises 25 items, each with three possible answers (not applicable, partially applicable, and clearly applicable) and is divided into five categories for emotion, conduct problems, hyperactivity/inattention, peer problems and prosociality. Each category comprises of five questions. A minimum score of 0 and a maximum score of 40 points can be achieved in the total difficulties score. For subscales, the range is from 0 to 10. Higher values indicate higher levels of difficulties in the problem subscales; the prosocial scale is reversed. In this study, only the proxy (parental) assessment in German language was used. For the total difficulties score, the following categorization was used: normal (score 0–13), borderline (score 14–16), and abnormal (score 17–40) difficulties (34).

## Ethical considerations

The study protocol of KRANIOPHARYNGEOM 2007 was proven and accepted by the ethical committee of the University of Würzburg, Germany; the protocol of KRANIOPHARYNGEOM Registry 2019 by the ethical committee of the Carl von Ossietzky University Oldenburg, Germany. The studies comply with the Declaration of Helsinki. All custodians were informed according to the study protocols and gave informed consent.

## Cases of perinatally diagnosed congenital craniopharyngioma

### Case 1

**Figure 1:**
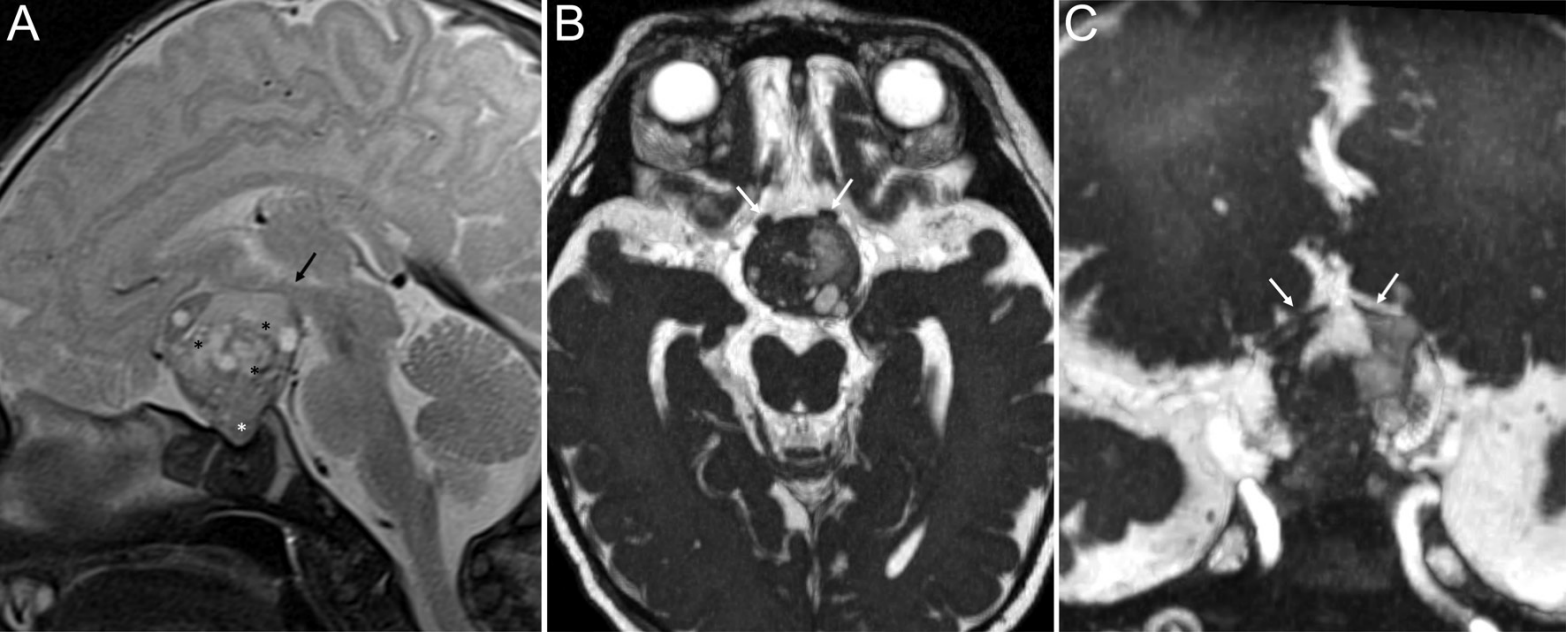
Magnetic resonance imaging (MRI) of a patient with perinatally diagnosed congenital craniopharyngioma (case 1). MRI at the fourth day of life demonstrating a cCP of intermediate size having more solid than cystic parts. The main part is in suprasellar location (A: black asterisk), only a small part is reaching intrasellarly (A: white asterisk). The optic chiasm and optic nerves are elevated and compressed (B and C: white arrows) and the posterior part is compressing the region of the mammillary bodies (A: black arrow) indicating an involvement of the posterior hypothalamus (A: sagittal T2 WI, sagittal and high resolution 3D-T2 WI with B: axial and C: coronal reconstruction).

Ultrasonography revealed a suprasellar tumor in a fetus at 33 weeks of gestation. The suprasellar mass was confirmed by prenatal MRI at 34 weeks of gestation (Fig. 1). The male newborn, delivered after 41 weeks of gestation, was operated at the age of 23 days, and an adamantinomatous CP was partially resected (Table 1). Postoperatively, pituitary deficiency and a transient syndrome of inappropriate antidiuretic hormone secretion (SIADH) were present (Table 2). At an age of 17 months, due to progression of residual tumor, a second partial resection was performed. A second progression has been documented at an age of 3.1 years. Therefore, a percutaneous proton beam therapy (total target volume dose of 54.9 Gy, fractions of 1.8 Gy) at an age of 3.4 years was applied. At a current age of 4.3 years, the patient showed a normal physical, mental, statomotor, and linguistic development with a body height of 111.1 cm (99 percentile) and a body weight of 20.5 kg (96 percentile) at last follow-up (Table 2). Since his first tumor resection, the patient continuously receives substitution with hydrocortisone, thyroxine and growth hormone. At a follow-up evaluation 4 years after cCP diagnosis, the total difficulties (SDQ) score was 31 for this child, which indicates an abnormal level of difficulties. For conduct problems and peer problems, the parents gave 9 and 6 out of 10 points, respectively. Regarding the emotional and hyperactivity/inattention domain, this child received 7 and 9 out of 10 points. In prosociality, the child received 4 points, indicating low levels of prosocial behavior (Table 2). The parents rated the FMH at percentile 10, indicating an impaired functional capacity for the child’s age.

**Table 1:** Characteristics of three patients perinatally diagnosed with congenital adamantinomatous craniopharyngioma (cCP) and recruited between 2007 and 2019 in the craniopharyngioma studies (KRANIOPHARYNGEOM 2007 and KRANIOPHARYNGEOM Registry 2019).

	Case 1	Case 2	Case 3
Sex	Male	Male	Female
Gestational complications	–	–	–
Gestational age at birth, weeks	41	37 + 2	37 + 2
Mode of delivery	Spontaneous	Cesarean section	Cesarean section
Birth weight, g (P) (28)	3625 (50)	3500 (75)	3330 (75)
Birth height, cm (P) (28)	54 (50)	50 (50)	48 (25)
Head circumference at birth, cm (P) (28)	36.0 (50)	35.0 (75)	39.5 (97)
Diagnostic method for primary diagnosis	Prenatal sonography	Postnatal sonography	Prenatal sonography
Age/GA at diagnosis	33 weeks of GA	Second day of life	16 weeks of GA
Tumor volume on MRI at diagnosis, cm³	5.29	1.06	74.84
Signs of hydrocephalus on MRI	No	No	Yes
Tumor location	Intra- and suprasellar	Intra- and suprasellar	Intra- and suprasellar
Age at first surgery (histological diagnosis), day of life	23	51	11
Surgical complications	–	–	–
Degree of surgical resection	Incomplete	Complete	Incomplete
Surgical approach	Right frontotemporal	Right subfrontal	Right frontal

GA , gestational age; P, percentile.

**Table 2:** Outcome of three patients perinatally diagnosed with congenital adamantinomatous craniopharyngioma (cCP) and recruited between 2007 and 2019 in the craniopharyngioma studies (KRANIOPHARYNGEOM 2007 and KRANIOPHARYNGEOM Registry 2019).

	Case 1	Case 2	Case 3
Follow-up interval, years	3.6	2.2	1.9
Age at last visit, years	3.6	2.1	2.0
Head circumference at last visit, cm (P) (30)	36 (age: 3 years; <3 P)	39 (age: 2 months; 25 P)	54.5 (age: 26 months; >99 P)
Height at last visit, cm (P) (1)	111.1 (99)	87.9 (25)	75 (0.1)
Weight at last visit, kg (P) (1)	20.5 (96)	12.8 (52)	9.6 (1)
Body mass index (s.d.s) (2) at last visit	+0.73	–0.03	+0.95
Presurgical grade of HI (3)	II	I	II
Grade of surgical HL (3)	I	I	0
Irradiation	Proton beam therapy	No	No
Arginine–vasopressin deficiency	No	Yes	No
TSH deficiency	Yes	Yes	No
ACTH deficiency	Yes	Yes	No
Gonadotropin deficiency	No	Yes	No
Growth hormone deficiency	Yes	Yes	Yes
Neurological sequelae	Normal psychomotor development	Normal psychomotor development	Psychomotor retardation, left hemiparesis
FMH, percentile (4)	10	25	3
SDQ, score (5)	31	17	9

ACTH, adrenocorticotropic hormone; FMH, Functional capacity scale Fertigkeitenskala Münster-Heidelberg; HI, hypothalamic involvement; HL, surgical hypothalamic lesion; n.a., not available; P, percentile; SDQ, The Strengths and Difficulties Questionnaire; TSH, thyroid-stimulating hormone.

### Case 2

**Figure 2:**
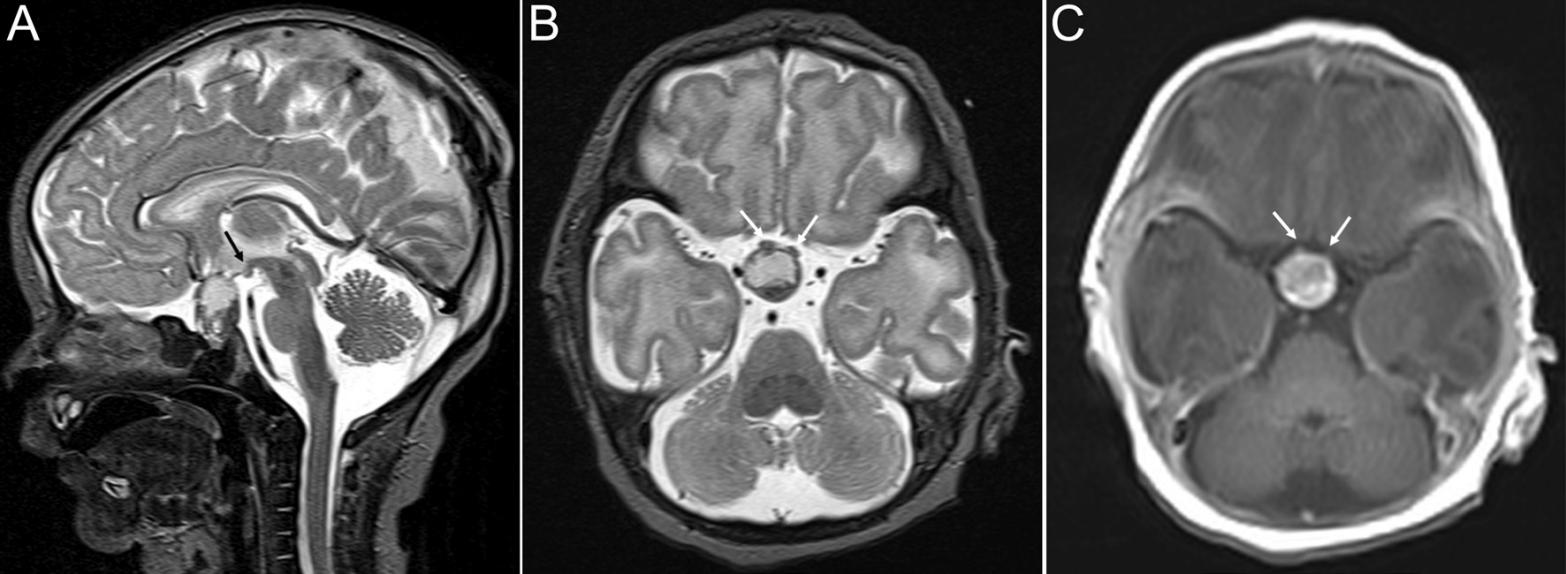
MRI of a patient with perinatally diagnosed congenital craniopharyngioma (case 2). The MRI obtained on the third day of life is displaying a small cCP with solid and cystic parts. About one-third is in intra- two-thirds in suprasellar location. The mammillary body (A: black arrow) is not affected, indicating that compression is limited to the anterior hypothalamus. Little compression and displacement of the optic nerves (B and C: white arrows) (T2 WI A: sagittal plane, B: axial plane, C: T1 WI with contrast enhancement).

The male newborn, delivered via cesarean section at 37 + 2 weeks of gestation (APGAR 9/9/9), developed postnatally a spontaneous pneumothorax (Table 1). Routine ultrasonography of the skull at the second day of life revealed a suprasellar tumor, which was confirmed by MRI 24 h later (Fig. 2). The tumor showed rapid progression (Fig. 2). A complete tumor resection was achieved via frontal approach at an age of 51 days and radiologically confirmed (Table 1). The histological diagnosis was adamantinomatous CP. The patient developed complete pituitary deficiency and is under endocrine treatment. At a follow-up evaluation 2.2 years after cCP diagnosis, the parents rated the FMH at percentile 25, indicating that the functional capacity is reduced in this child (Table 2). In terms of strengths and difficulties (SDQ score), the total difficulties score was 17 for this child, which indicates an abnormal level of difficulties. For conduct problems and peer problems, the parents gave 6 and 7 out of 10 points, respectively. Regarding the emotional and hyperactivity/inattention domain, this child received 0 and 4 out of 10 points. In prosociality, the child received 4 points, indicating low levels of prosocial behavior (Table 2).

### Case 3

**Figure 3:**
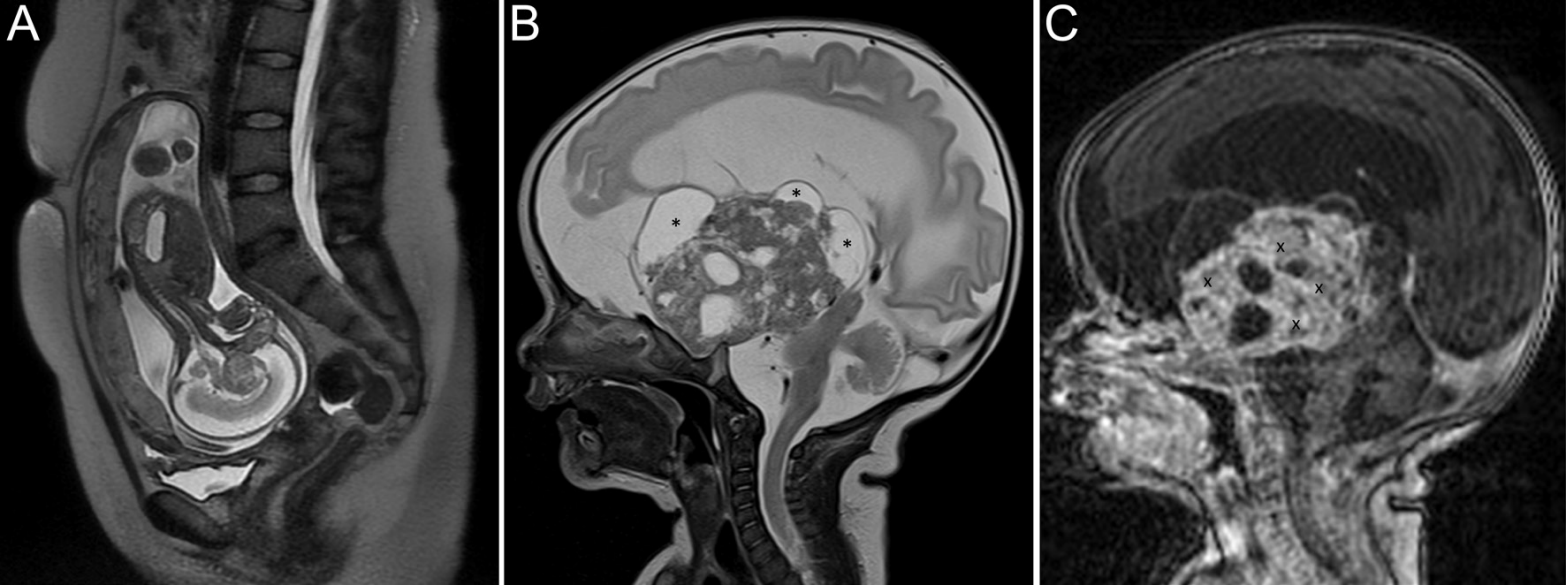
MRI of a patient with perinatally diagnosed congenital craniopharyngioma (case 3). Intrauterine MRI made at 16 weeks of gestation (A) with sagittal T2-weighted image (WI) showing a sagittal oblique plane of the fetus having a suprasellar mass in an early developmental stage of brainstem, cerebellum and cerebrum. MRI on the 15. day of life displaying growth of the cCP in all directions with huge cystic (B: black asterisks) and contrast-enhancing solid parts (C: black x’s). The brainstem, anterior, and posterior hypothalamus are compressed and displaced (B: sagittal T2WI and C: sagittal T1 WI with contrast enhancement).

**Figure 4:**
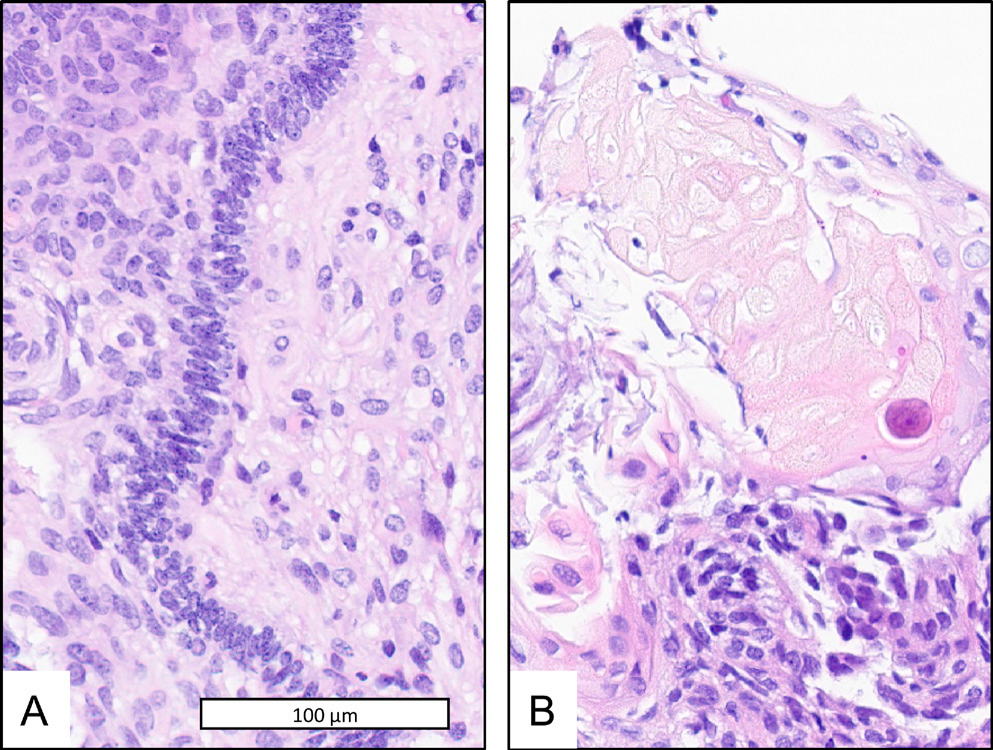
Hematoxylin and eosin staining of tumor material of case 3 showing palisading of the basal cells in panel A and wet keratin with ghost cells in panel B.

Routine ultrasonography revealed a suprasellar tumor in a fetus at 16 weeks of gestation. The suprasellar mass was confirmed by prenatal MRI at 16 weeks of gestation (Fig. 3). The female newborn, delivered after 37 weeks of gestation (APGAR 8/9/10), was operated at an age of 11 days. A subtotal resection was achieved. Biopsies confirmed an adamantinomatous CP and a ventriculoperitoneal shunt was inserted. Severe infections of the ventriculoperitoneal shunt had to be treated antibiotically and by intermittent external drainage of cerebrospinal fluid. The patient developed a left-sided hemiparesis and visual impairment. Due to the macrocephaly, she required specialized care. The girl developed growth hormone deficiency. Since the age of 9 months, her growth curve had stayed under the lowest percentile (0.1 percentile). At follow-up (age: 2.5 years), she was of 75.0 cm height. Her BMI s.d.s. was +0.95. She was unable to sit but was able to listen to stories and songs. Her parents rated the FMH at percentile 3, indicating a low functional capacity in her age group. For strengths and difficulties, the patient received a total score of 9, indicating a normal amount of difficulties. In the emotional, hyperactivity/inattention, conduct problems, and peer problems domains, she received 0, 4, 1, and 4 out of 10 points, respectively. Noteworthy among the results is that she scored 2 points in the prosociality domain, indicating low prosocial behavior. Figure 4 shows the classical pathology of an adamantinomatous CP of this patient.

## Discussion

Congenital CP is a very rare perinatal diagnosis, with a prevalence of 0.83% in our overall cohort. About 0.5–1.9% of all pediatric brain tumors are congenital brain tumors (35). Teratoma, primitive neuroectodermal tumor (PNET), astrocytoma and papilloma of the choroid plexus are the most commonly mentioned congenital central nervous system tumors in the literature (7). These congenital tumors are often located in supratentorial regions of the brain in contrast to tumors developing later in life.

Pediatric adamantinomatous CPs are diagnosed with a median age at diagnosis of 5–9 years and are located in the sellar and suprasellar region. The exact etiology and the reason for this age peak at CP diagnosis are unknown. Molecular changes in exon 3 of the β-catenin gene are associated with the adamantinomatous CP (36). The tumor can be present already at the time of birth.

We have recently analyzed the so far largest (*n* = 709) published cohort of patients after diagnosis of childhood-onset adamantinomatous CP in terms of clinical presentation and outcome and asked the question, whether age at diagnosis of adamantinomatous CP influences clinical presentation, therapy, and outcome. Patients diagnosed at an age <2 years presented with reduced functional capacity as measured by FMH scale, when compared to CP patients diagnosed during early childhood or school age. We reported that not only for the subgroup of patients younger than 6 years of age at CP diagnosis but also in the older age groups (at CP diagnosis), younger age was a risk factor for events in terms of progression and relapses. On the other hand, overall survival rates were similar in all age subgroups, indicating that radio-oncological treatment and/or surgical reinterventions were efficient rescue treatment modalities in case of relapse or progression of CP (22).

In our current study, we analyzed three of 361 cases with childhood-onset CP, recruited in the studies KRANIOPHARYNGEOM 2007 and KRANIOPHARYNGEOM Registry 2019 and diagnosed pre- and perinatally, with regard to clinical presentation, treatment, and outcome. With an estimated incidence rate of 1 patient per 1 million persons per year (7), around 480 newly diagnosed cases with childhood-onset CP could be expected over a period of 20 years in Germany. Accordingly, the estimated prevalence rate of 0.83% is representative for the very rare condition in Germany.

After follow-up, results on functional capacity (FMH) and strength and difficulties (SDQ) were compared for the different cases of cCP. The functional capacity was reduced in all three patients (FMH percentile 25, 10, and 3), showing lower skills and abilities compared to healthy peers. In terms of strengths and difficulties, two of the three cases had an abnormal total difficulties score. For case 1, parents reported difficulties on all domains and a low prosociality. The patient presented with presurgical anterior and posterior involvement of the hypothalamus (HI grade II). The tumor was resected incompletely under protection of the posterior hypothalamus (HL grade I). The parents of case 2 reported difficulties in the behavioral domains (conduct problems and peer problems); the other domains were normal. This case 2 presented with presurgical involvement of the anterior hypothalamus (HI grade I). The CP with anterior HI could be resected completely with preservation of the integrity of the posterior hypothalamus (HL grade I). For case 3, the parental assessment indicated a normal total difficulties score. Only the prosociality score was reduced in this child. Due to her psychomotor retardation, the girl was limited in her mobility and (peer) interaction. Therefore, many questions of the strength and difficulties questionnaire are not applicable to describe the current situation of the child.

Behavioral changes and difficulties are often consequences of hypothalamic dysregulation. Possible behavioral problems include impulse-control disorder, hoarding, and rage (15). These difficulties might directly interfere with the conduct and peer problem domain of the SDQ.

Reviewing the literature on cases with prenatal and perinatal diagnosis of cCP (Table 3) shows, that this age period is exceptional for diagnosis of CP and that the results of surgery in these patients have been poor, considering the reported mortality and morbidity rates. Summarized in Table 3, 12 cases with prenatal and perinatal diagnosis of cCP have been reported in the literature since 1985. Five of the reported cases in the literature died perinatally and nine cases died during the first year of life. The most recent case of Kageji *et al.* survived after a follow-up of 4 years (8). In our reported cases – most of them recently diagnosed (year at diagnosis: case 1: 2018; cases 2 and 3: 2020) – prognoses seem to be better with regard to morbidity and mortality. In the literature, congenital CPs are usually described as large-sized tumors with suprasellar extension, causing a compression of the optic chiasm. Also, one of our cases had a very large-sized tumor (74.84 cm³). In terms of surgery, four cases in the literature had no surgical intervention at all. All of our cases were successfully surgically treated during the first 2 months of life, one patient with complete resection, and the other two patients with incomplete resection. One patient with incomplete resection received proton beam therapy at the age of 3 years. Psychomotor development was normal after proton beam therapy, whereas psychomotor retardation and neurological deficits occurred in the case characterized by large tumor volume, hydrocephalus and posterior hypothalamic involvement (case 3).

**Table 3:** Published cases (since 1985) of congenital craniopharyngioma diagnosed perinatally.

Reference	Sex	GA (weeks)	Delivery mode	Time of diagnosis (GA, weeks)	Tumor size at diagnosis (MRI)	Hydrocephalus	Tumor location	First OP (age, surgical approach, degree of resection)	Survival/outcome
Lonjon *et al.* 2005 (37)	Male	34	spo	29	32 × 23 × 22 mm 3D: 8.10 cm³	No	Suprasellar	40 days, subfrontal GTR	1 year, good condition
Kültürsay *et al.* 1995 (38)	n.a.	n.a.	sec	29	25 mm diameter	n.a.	Suprasellar	4 weeks	Died during surgery
Müller-Scholden *et al.* 2000 (39)	Male	38	spo	28	50 mm diameter	No	Suprasellar	17 days, pterional GTR	8 years, hemiplegia
Jurkiewicz *et al.* 2010 (8)	n.a.	36	sec	28	40 × 35 × 34 mm 2D: 14 cm² 3D: 23.8 cm³	Yes	SS	4 weeks, GTR	3.5 months
Do Prado Aguiar *et al.* 2013 (40)	Male	38	sec	29	68 × 66 × 62 mm 3D: 139.13 cm³	Yes	Suprasellar	14 days, transventricular PR	Died 8 months after surgery
Kageji *et al.* 2017 (41)	n.a.	40	sec	37	16 × 22 × 15 mm 3D: 2.64 cm³	No	Supra-sellar	3 months, interhemispheric, GTR	4 years, normal physical and mental development
Freeman *et al.* 1988 (42)	Female	35	sec	35	12 × 8 × 1 0 mm 3D: 0.48 cm³	No	Suprasellar	No surgery	Died second day of life
Arai *et al.* 2003 (43)	Female	40	spo	33	32 × 28 × 30 mm 3D: 13.44 cm³	Yes	Suprasellar	9 months GTR	6 years, physical and mental development almost normal
Gongidi *et al.* 2012 (44)	Male	23	spo	23	55 mm diameter	Yes	Suprasellar	No surgery	Stillborn
Sosa-Olavarría *et al.* 2000 (45)	n.a.	21	Abortion	20	n.a.	No	Supra-sellar	No surgery	Abortion
Synder *et al.* 1986 (46)	Female	35	sec	35	n.a.	n.a.	n.a.	No surgery	Died third day of life
Bailey *et al.* 1990 (47)	Male	36	sec	27	n.a.	Yes	Suprasellar	9 days VP-Shunt	Died at 8 weeks of life
Wellons *et al.* 2006 (48)	Male	40	spo	First day of life	80 × 60 × 70 mm 3D: 168.00 cm³	n.a.	Suprasellar	8 days, pterional, PR	1 year, healthy and vigorous, residual right-sided third cranial nerve palsy

sec, cesarean section; spo, spontaneous delivery.

This prognostic improvement might be related to progress in diagnostic and therapeutic methods especially with regard to modern MRI, neurosurgical and radio-oncological techniques as well with regard to close endocrinological and neurological monitoring. We hypothesize, that a hypothalamus-sparing surgical approach results in better outcome for the children, especially diagnosed with CP at this early age. All of our reported cases presented with a hypothalamus-involving tumor presurgically (Table 2). None of our cases had a posterior hypothalamic lesion (HL grade II) after surgery, indicating hypothalamus-sparing surgical strategies. Future research should aim to assess the benefits and risks of radiation therapy applied in patients of young age.

We conclude, based on improvements of diagnostic methods and therapeutic approaches, earlier prenatal diagnosis of cCP should lead to intrauterine transferal of cCP patients to a specialized center for postnatal treatment of newborns with sellar masses by a multidisciplinary team to secure an improved prognosis and long-term outcome of these patients.

## Declaration of interest

H.L.M. has received reimbursement of participation fees for scientific meetings and continuing medical education events from the following companies: Ferring, Pfizer, Sandoz/Hexal, Novo Nordisk, IPSEN, and Merck Serono. He has received reimbursement of travel expenses from Merck Serono and IPSEN and lecture honoraria from Pfizer. G.B. has received honoraria for lectures from Ferring, Ipsen, Lilly, Merck, Novo Nordisk, Pfizer, and Sandoz as well as for membership in advisory boards of Ferring, Ipsen, Merck, Novo Nordisk, Pfizer, Ascendis, and Sandoz. G.F. has received reimbursement fee for meetings in advisory boards from Novartis. B.B. has received honorarium for lecture from Merck Serono. K.S. has received honoraria for lectures and membership in advisory boards as well as reimbursement fees for meetings from Servier, Jazz Pharmaceuticals, Novartis, Roche, Bayer, and NovoNordisk. The other authors declared no conflict of interest.

## Funding

This study was funded by grants (H.L.M., DKS2014.13; B.B., DKS2018.02) of the German Childhood Cancer Foundation, Bonn, Germany.

## Data availability statement

The datasets generated during the current study are available from the corresponding author on reasonable request.

## Author contribution statement

J.B. researched the data and wrote the manuscript. S.B. collected the data, contributed to the analytical plan and discussion and reviewed/edited the manuscript. K.S. collected the data, contributed to the analytical plan and discussion and reviewed/edited the manuscript. F.H.S. collected the data, contributed to the analytical plan and discussion and reviewed/edited the manuscript. G.F. collected the data, contributed to the analytical plan and discussion and reviewed/edited the manuscript. G.B. collected the data, contributed to the analytical plan and discussion and reviewed/edited the manuscript. B.B. did neuroradiological assessment of all imaging. B.B. is the neuroradiologist, who performs reference-assessment of imaging in all patients recruited in KRANIOPHARYNGEOM 2007 and KRANIOPHARYNGEOM Registry 2019. She prepared the imaging data and their presentation and reviewed/edited the manuscript. T.P. performed reference pathology assessments. He prepared the histological figure and reviewed/edited the manuscript. C.F. contributed to the analytical plan and discussion and reviewed/edited the manuscript. H.L.M. initiated and conducted the multicenter trials KRANIOPHARYNGEOM 2007, and KRANIOPHARYNGEOM Registry 2019. H.L.M. designed the analytical plan of the study and discussion and reviewed/edited the manuscript.
